# Pseudolymphoma of the nipple treated with topical imiquimod and cryotherapy

**DOI:** 10.1016/j.jdcr.2022.10.008

**Published:** 2022-10-15

**Authors:** Amy Xiao, John D. Miedler, Oleg E. Akilov

**Affiliations:** a School of Medicine, University of Pittsburgh, Pittsburgh, Pennsylvania; c Cutaneous Lymphoma Program, Department of Dermatology, University of Pittsburgh, Pittsburgh, Pennsylvania; b Dermpath Diagnostics, Pittsburgh, Pennsylvania

**Keywords:** atrophy, imiquimod, lymphoproliferative disorder, nipple, pseudolymphoma, sclerosis, treatment

## Introduction

Pseudolymphoma is difficult to treat because the external trigger is frequently unknown, and even if it is known, removal does not guarantee a cure. There is a lack of standardized treatment, but generally accepted options for the treatment of pseudolymphoma include observation, antibiotics, topical, intralesional and systemic corticosteroids, cryosurgery, photochemotherapy, local radiation therapy, and surgical excision.^1^ Treatment choice should depend on patient comfort, expectations, and the location of the lesion because some areas of the skin are more delicate than others. Here, we present a case of pseudolymphoma of the nipple, which presented a challenge in terms of treatment because of its location, successfully treated with topical imiquimod and cryotherapy.

## Case report

**Fig 1:**
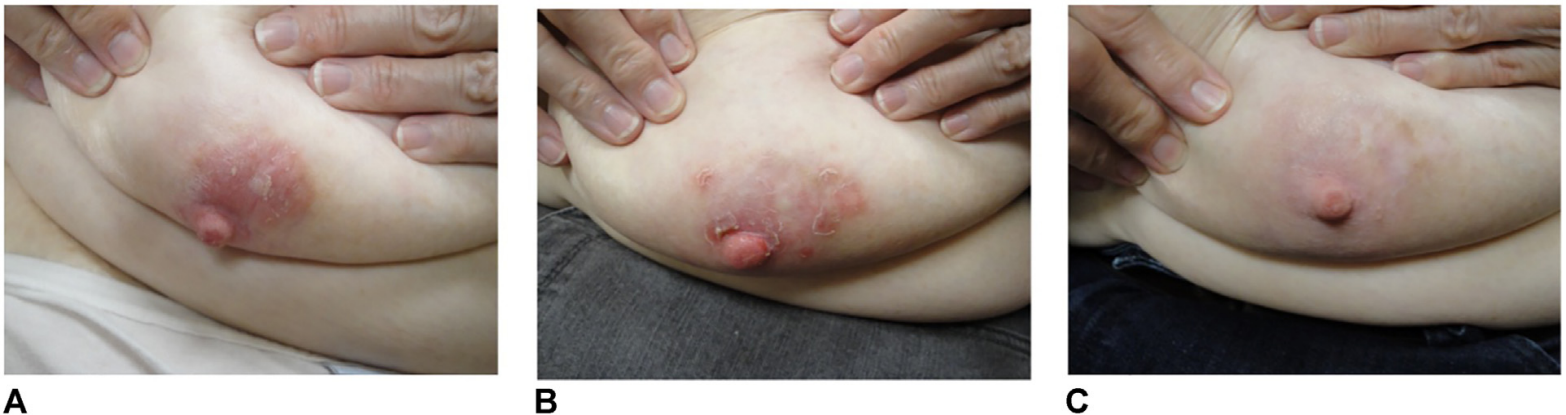
Pseudolymphoma on the right nipple. **A,** Before the treatment, **B,** 42 weeks after treatment, and **C,** 57 weeks after treatment.

**Fig 2:**
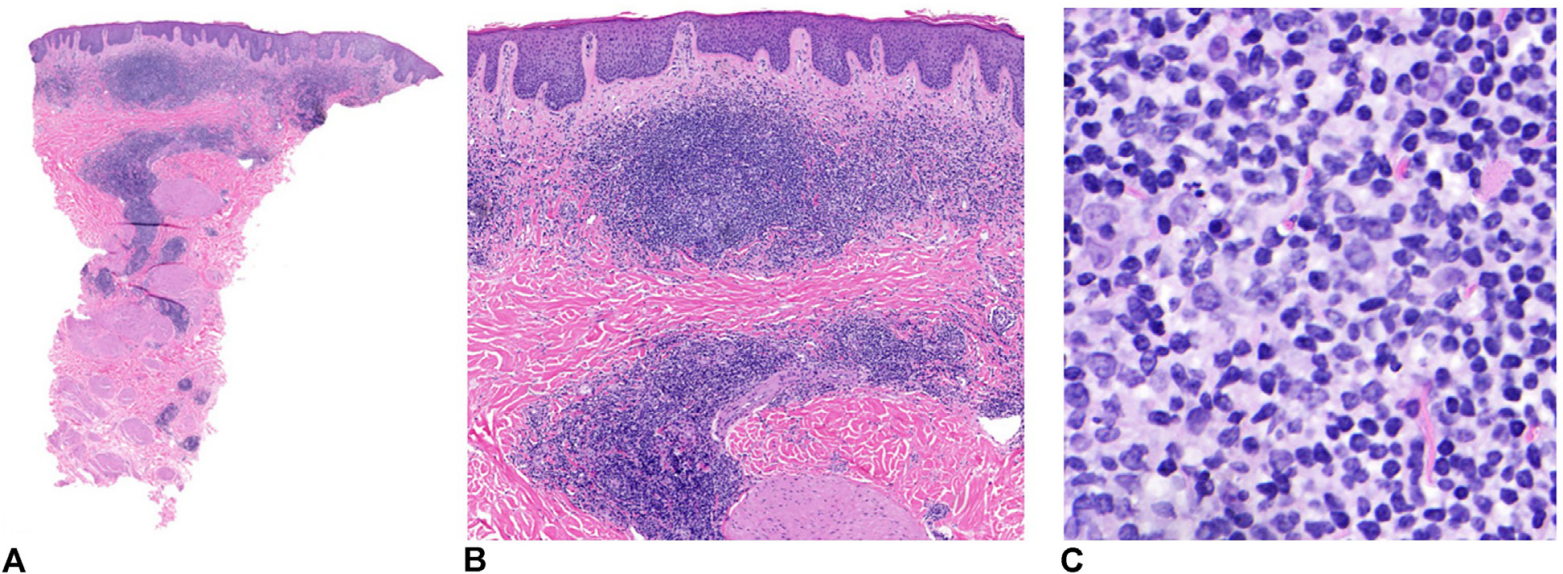
Punch biopsy of the right nipple. Dermal papillary and reticular nodular infiltrates of lymphocytes with an admixture of eosinophils and histiocytes. (**A, B,** and **C,** Hematoxylin-eosin stain; original magnifications: **A,** ×10; **B,** ×20; and **C,** ×40.)

**Fig 3:**
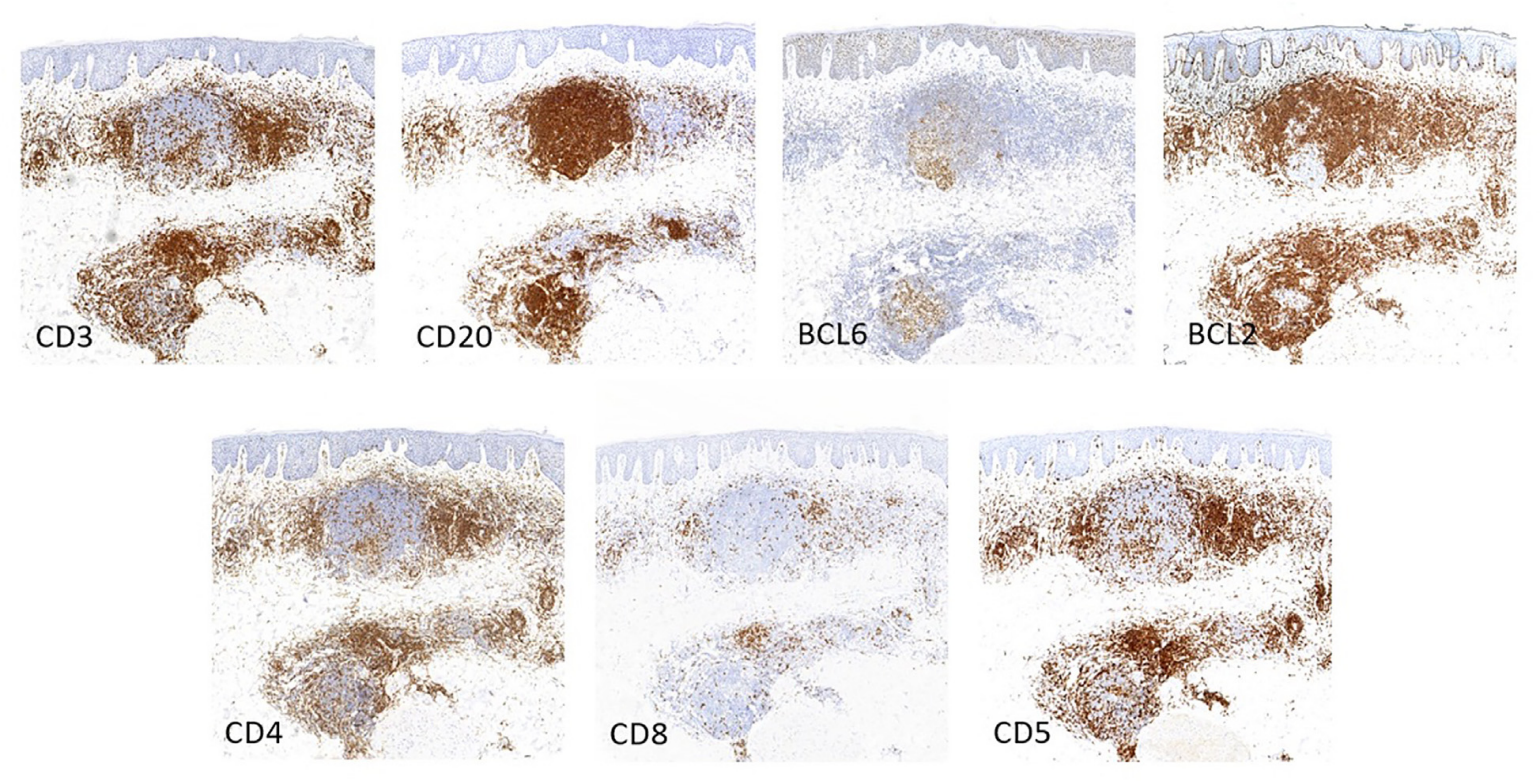
Immunohistochemical staining highlighted the nodular infiltrate of CD20^+^ B-cell lymphoma2^+^ lymphocytes with CD4^+^ T cells on the periphery.

A 78-year-old woman was evaluated for chronic bilateral nipple eruption of 5 to 6 years’ duration, believed to be because of irritation from her bra. Physical examination showed well-demarcated 2 cm in diameter nummular mildly pruritic erythematous plaque with a fine scale on the left and right upper external quadrant of both nipples (Fig 1). The plaques persisted despite treatment with topical mild-to-moderate corticosteroids. The specimen demonstrated a nodular and diffuse inflammatory cell infiltrate within the dermis (Fig 2, *A* and *B*). The inflammatory cell infiltrate was comprised predominantly of small-to-medium size lymphocytes, but there were also intermingled eosinophils and histiocytes (Fig 2, *C*). Immunohistochemical staining for CD3 and CD20 showed an admixture of CD3^+^ T cells and CD20^+^ B cells. The CD20^+^/CD5^−^/CD10^−^ B cells showed expression predominantly within the nodular aggregates (lymphoid germinal center with retained mantle zones) with a retained expression of B-cell lymphoma 6 and no aberrant expression of B-cell lymphoma 2. CD3^+^ T cells show an appropriate CD4:CD8 with a retained expression of CD5 and CD7 (Fig 3). The systemic lymphoid process was excluded by imaging and complete blood cell count. The lesions were treated with cryotherapy monthly and imiquimod 5% cream 3 times per week (Fig 1). They were stable 2 months later but had approximately 90% clearance in the third month. The total treatment duration was 4 months. Side effects of the treatment included minimal irritation and bleeding around the nipple when showering.

## Discussion

The nipple is a difficult skin area to treat because injections of corticosteroids can cause subcutaneous fat atrophy, and radiation therapy to the breast has been documented to induce dermal sclerosis, dimpling, and lymphedema.^2^^,^^3^ To our knowledge, this is the first report of topical imiquimod as an effective treatment for pseudolymphoma. Imiquimod is an immunomodulator^4^ that binds to toll-like receptor 7 expressed in antigen-presenting cells, which induce innate and adaptive immunity and stimulates cytokine production.^5^ It is approved for the treatment of anogenital warts, actinic keratosis, and superficial basal cell carcinoma, and is used off-label for the successful treatment of numerous conditions, including squamous cell carcinoma, penile and vulvar intraepithelial neoplasia, molluscum contagiosum, and lentigo maligna.^4^^,^^5^ Documentation of this patient’s presentation and favorable clinical course with topical imiquimod with topical imiquimod will offer another therapeutic approach to pseudolymphoma, especially for delicate skin areas.

## Conflicts of interest

None disclosed.
